# Giant intramuscular lipoma of the tongue: a case report and literature review

**DOI:** 10.4076/1757-1626-2-7906

**Published:** 2009-06-22

**Authors:** Giuseppe Colella, Paolo Biondi, Rosario Caltabiano, Giada Maria Vecchio, Paolo Amico, Gaetano Magro

**Affiliations:** 1 Department of Head and Neck Surgery, Second University of Naples, Naples, Italy; 2 Department G.F. Ingrassia, Section of Anatomic Pathology, University of Catania, Catania, Italy

## Abstract

We herein report a rare case of giant intramuscular lipoma of the tongue. A 75-year-old Italian male presented at our department with a large tumor at the tip of the tongue that had been present for over 30 years. Clinical examination revealed a yellowish lesion, measuring 10 cm in maximum diameter, protruding from lingual surface. Histological examination showed an unencapsulated lipomatous tumor composed of mature adipocytes, uniform in size and shape, diffusely infiltrating striated muscle fibers of the tongue. The patient is well with no local recurrence after a 15-month follow-up period.

## Introduction

Lipoma is the most common benign soft tissue mesenchymal neoplasm [1]. Histologically, lipoma can be classified as conventional lipoma, fibrolipoma, angiolipoma, spindle cell/pleomorphic lipoma, myxolipoma, chondroid lipoma, osteolipoma and myolipoma, [1]. Occasionally (1-4% of cases) lipoma can occur in the oral cavity [2]-[4], including tongue where it usually presents as long-standing soft nodular asymptomatic swellings covered by normal mucosa [4]. Intramuscular lipoma, also known as *infiltrating lipoma*, is a slowly-growing painless lesion typically found in the large muscles of the extremities of adult males, usually characterized by diffuse infiltration of striated muscle fibers [5]-[6]. We report an unusual case of giant intramuscular lipoma of the tongue, emphasizing the clinico-pathologic features and differential diagnostic problems.

## Case presentation

**Figure 1:**
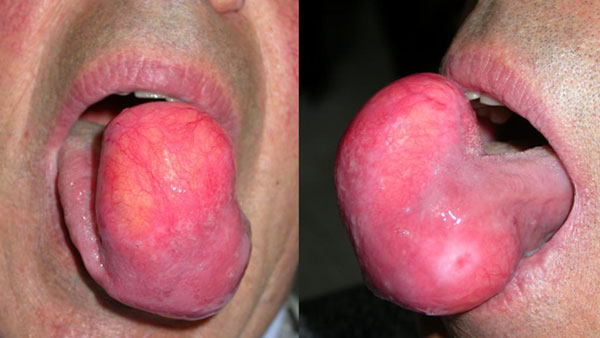
**Clinical examination showing a giant, yellowish, submucosal mass involving tongue**.

A 75-year-old Italian male presented at our department with a large tumor at the tip of the tongue that had been present for over 30 years. The patient denied neurosensory disturbance, including dysgeusia. His speech was not very clear due to the bulkiness of the mass and he had difficulties for swallowing. Clinical examination revealed a yellowish lesion, measuring 10 cm in maximum diameter, protruding from lingual surface and covered by mucosa rich in vessels (Figure 1). On palpation, the lesion was rubbery and not fluctuant. Computer tomography (CT) scan revealed a poorly circumscribed lesion exhibiting a high density, consistent with lipomatous tissue. The diagnosis of lipoma was proposed, accordingly. A transoral V-shaped surgical excision was performed under loco-regional anesthesia. Sutures were removed on the 10^th^ postoperative day. The specimens were fixed in 10% buffered formalin, and embedded in paraffin for routine histological examination.

**Figure 2:**
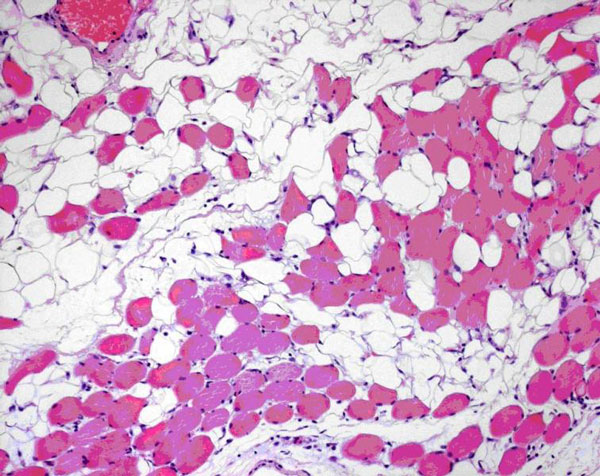
**Histological examination showing mature adipocytes diffusely infiltrating striated muscles**.

Histological examination (hematoxylin and eosin stained slides) showed an unencapsulated lipomatous tumor composed of mature adipocytes, uniform in size and shape, diffusely infiltrating striated muscle fibers of the tongue (Figure 2). Lipoblasts, cytologic atypia, mitoses or necrosis were not observed. Surgical margins were tumor-free. The patient is well with no local recurrence after a 15-month follow-up period.

## Discussion

World Health organization (WHO) classification of benign lipomatous tumors recognizes conventional lipoma, fibrolipoma, angiolipoma, spindle cell/pleomorphic lipoma, myxolipoma, chondroid lipoma, osteolipoma, myolipoma, lipomatosis, lipomatosis of nerve, lipoblastoma/lipoblastomatosis, and hibernoma [1]. Lipomas are the most common mesenchymal tumors of soft tissue, but they are relatively uncommon in the oral and maxillofacial region [7], being no more of 4% of the tumours occurring in this sites [8]. Buccal and tongue mucosa are the most frequent sites, followed by the floor of the mouth, buccal vestibule, palate, lips, and gingiva [7]. Although a male predominance has been reported [4], some authors found an equal gender distribution for oral lipomas [3].

Intramuscular (infiltrating) lipoma is a rare variant of lipoma first defined by Regan and his colleagues in 1946 [9]. Clinically, oral intramuscular lipoma presents as a well circumscribed painless, solitary, rubbery, submucosal swelling. Although it arises in the deeper tissues of tongue [6], a protrusion from the lingual mucosa can be documented in most large-sized lesions [10]-[12]. We report an unusual case of a giant lipomatous lesion of the tongue which fulfilled all the histological criteria of intramuscular lipoma. Tumor presented as a single lesion measuring 10 cm in its greatest diameter, slowly growing over a period of 30 years. Clinical course of oral intramuscular lipomas is usually asymptomatic, but on rare occasions, the infiltration is so extensive that it can cause muscle dysfunction or sensory changes due to pressure on nerve trunks [5,13]. In the present case the large size of the tumor interfered either with speech or swallowing. On CT scan, intramuscular lipoma shows a high density from 83 to 143 Hamsfield units with poorly defined margins. The magnetic resonance imaging can be useful. The strikingly high intensity signals on both T1- and T2-weighted images are suggestive of lipoma, and it further delineates the extent of tumour involvement [14]. In our case, a preoperative diagnosis of lipoma was suggested on CT scan features. Treatment of intramuscular lipoma is based on its complete surgical excision, whenever possible. The typical infiltrative growth pattern of intramuscular lipoma can be responsible of a potential misdiagnosis of malignancy. Differential diagnosis of intramuscular lipoma mainly revolves around liposarcoma. Unlike the latter, however, the former lacks lipoblasts, cellular pleomorphism, marked vascularisation and mitotic activity [15]. In our case, although adipocytes infiltrated striated muscle fibers, they were all mature and uniform in shape and size. In addition, no morphological worrisome feature of liposarcoma, previously mentioned, could be identified. Local recurrence of intramuscular lipoma has been observed in up 62.5% of the cases [16]. This high rate of recurrence is probably related to the difficulties in achieving radical surgical excision. After surgical treatment no recurrences of giant intramuscular lipoma are reported in the literature [10]-[12]. In our case, patient is well with no recurrence after a 15-month follow-up period. No future local recurrence is expected as a complete tumor excision was obtained.

## Abbreviations

CT: Computer tomography; WHO: World Health organization.

## Consent

Written informed consent was obtained from the patient for publication of this case report and accompanying images. A copy of the written consent is available for review by the Editor-in-Chief of this journal.

## Competing interests

The authors declare that they have no competing interests.

## Authors' contributions

GC and PB were major contributors in surgical excision of the lesion. GM and RC were major contributors in the histological diagnosis. GMV and PA were major contributors in writing the manuscript. All authors read and approved the final manuscript.
